# Mutations spectrum in hereditary disorders predisposing to occurrence of intestine polyposis in Poland

**DOI:** 10.1186/1897-4287-10-S4-A24

**Published:** 2012-12-10

**Authors:** Andrzej Plawski, Paweł Boruń, Tomasz Banasiewicz, Jacek Paszkowski, Agnieszka Stembalska, Maria Sąsiadek, Monika Siołek, Beata Kozak - Klonowska, Izabela Brozek, Janusz Limon, Dorota Nowakowska, Grzegorz Kurzawski, Tomasz Byrski, Tomasz Gach, Diana Hodorowicz-Zaniewska, Anna Bartkowiak, Ryszad Slomski, Elżbieta Czkwianianc, Krzysztof Linke, Ewa Grzybowska, Arleta Lącka-Wojciechowska, Marek Szwiec, Sabina Więcek, Alicja żabka, Agnieszka Synowiec, Anna Jakubiuk-Tomaszuk, Robert Skalski, Jan Lubinski, Piotr Krokowicz, Paweł Blecharz, Mikołaj Teisseyre, Michal Drews, Wojciech Cichy

**Affiliations:** 1 Institute of Human Genetics Polish Academy of Sciences, Poznan, Poland; 2 Genetic Counseling Unit, Holy Cross Cancer Center, Kielce, Poland; 3 Department of General, Gastroenterological and Endocrinological Surgery, University of the Medical Sciences, Poznan, Poland; 4 Department of Biology and Genetics, Medical University of Gdańsk, Poland; 5 Genetic Counseling Unit Cancer Center and Institute of Oncology, Warsaw, Poland; 6 Department of Genetics and Pathology, Pomeranian Medical University, Szczecin, Poland; 7 Department of Genetics, Medical University Wroclaw, Wroclaw, Poland; 8 The Children's Memorial Health Institute, Warsaw, Poland; 9 1st Department of General and GI Surgery, Jagiellonian University, Cracov, Poland; 10 Department of Pediatrics and Gastroenterology, Institute of Polish Mother’s Memorial Hospital, Lodz, Poland; 11 International Oncotherapy Centre, Koszalin, Poland; 12 Department of Gastroenterology Human Nutrition and Internal Diseases, K.Marcinkowski University School of Medical Sciences, Poznan, Poland; 13 Department of Tumor Biology, Centre of Oncology, Maria Sklodowska-Curie Memorial Institute, Gliwice, Poland; 14 Regional Oncology Center, Opole, Poland; 15 Upper- Silesia Child Health Center Katowice, Poland; 16 Medical University of Bialystok, Poland; 17 University of the Medical Sciences, Katowice, Poland; 18 Department of Surgery University of the Medical Sciences, Poznan, Poland; 19 MilitaryHospital,Warszawa, Poland; 20 Department of Pediatrics University of the Medical Sciences, Poznan, Poland

## 

The term polyp refers to any overgrowth of tissue from the surface of mucous membranes. Intestinal polyps grow out of the lining of the small and large bowels. The polyps that arise as a result of proliferative dysplasia are termed as adenomatous polyps or adenomas. They are true neoplastic lesions and are precursors of carcinoma. The hamartomatous polyps are formed as a result of abnormal mucosal maturation. They are non-neoplastic and do not have malignant potential. There are several hereditary diseases that produce large numbers of intestinal polyps. These disorders include: familial adenomatous polyposis of the colon (MIM 175100), familial adenomatous polyposis type 2(MIM 608456), Lynch's syndrome (MIM 120435), Peutz-Jeghers syndrome (MIM 175200), Juvenile polyposis syndrome (MIM 174900) PTEN Hamartoma Tumor Syndrome (PHTS) PHTS Includes: Bannayan-Riley-Ruvalcaba Syndrome (MIM 153480), Cowden Syndrome (MIM 153480), PTEN-Related Proteus Syndrome, Proteus-Like Syndrome. Here we present spectrum of mutation detected in over six hundred Polish families with intestinal polyposis. The studies have encompassed over 30 families with Juvenile polyposis syndrome and PHTS, over 40 families with Peutz-Jeghers syndrome and almost 600 families with familial adenomatous polyposis of the colon. The study was in part financed by the Ministry of Education and Science, Poland, grant number N402 481537, N401 331936.

